# Anti-CXCR4 Antibody Combined With Activated and Expanded Natural Killer Cells for Sarcoma Immunotherapy

**DOI:** 10.3389/fimmu.2019.01814

**Published:** 2019-08-02

**Authors:** Maria Vela, David Bueno, Pablo González-Navarro, Ariadna Brito, Lucía Fernández, Adela Escudero, Jaime Valentín, Carmen Mestre-Durán, Marina Arranz-Álvarez, Rebeca Pérez de Diego, Marta Mendiola, José Juan Pozo-Kreilinger, Antonio Pérez-Martínez

**Affiliations:** ^1^Translational Research in Pediatric Oncology, Hematopoietic Transplantation and Cell Therapy, Hospital La Paz Institute for Health Research (IdiPAZ), Madrid, Spain; ^2^Pediatric Hemato-Oncology Department, Hospital Universitario La Paz, Madrid, Spain; ^3^H12O-CNIO Hematological Malignancies Clinical Research Unit, Spanish National Cancer Research Centre (CNIO), Madrid, Spain; ^4^Molecular Pediatric Oncology Unit, Institute of Medical and Molecular Genetics (INGEMM), La Paz University Hospital, Madrid, Spain; ^5^Biobank of Hospital La Paz Institute for Health Research (IdiPAZ), Madrid, Spain; ^6^Laboratory of Immunogenetics of Human Diseases, Hospital La Paz Institute for Health Research (IdiPAZ), Madrid, Spain; ^7^Innate Immunity Group, Hospital La Paz Institute for Health Research (IdiPAZ), Madrid, Spain; ^8^CIBER of Respiratory Diseases (CIBERES), Madrid, Spain; ^9^Molecular Pathology and Therapeutic Targets, Hospital La Paz Institute for Health Research (IdiPAZ), Madrid, Spain; ^10^Molecular Pathology Section, Institute of Medical and Molecular Genetics (INGEMM), La Paz University Hospital, Madrid, Spain; ^11^Pathology Service, La Paz University Hospital, Madrid, Spain; ^12^Department of Pediatric, Universidad Aut ónoma de Madrid (UAM), Instituto de Investigaci ón Sanitaria del Hospital Universitario La Paz (IdiPAZ), Madrid, Spain

**Keywords:** sarcoma, metastasis, chemokine C-X-C receptor 4 (CXCR4), therapeutic antibody, activated and expanded natural killer (NKAE) cells, immunotherapy

## Abstract

Sarcoma is one of the most severe forms of pediatric cancer and current therapies -chemotherapy and surgery- fail to eradicate the disease in half of patients. Preclinical studies combining new therapeutic approaches can be useful to design better therapies. On one hand, it is known that CXCR4 expression is implicated in rhabdomyosarcoma progression, so we analyzed relapses and chemotherapy-resistant rhabdomyosarcoma tumors from pediatric patients and found that they had particularly high levels of CXCR4 expression. Moreover, in assays *in vitro*, anti-CXCR4 blocking antibody (MDX1338) efficiently reduced migration and invasion of alveolar rhabdomyosarcoma RH30 cells. On the other hand, activated and expanded natural killer (NKAE) cell therapy showed high cytotoxicity against sarcoma cells *in vitro* and completely inhibited RH30 tumor implantation *in vivo*. Only the combination of MDX1338 and NKAE treatments completely suppressed metastasis in mice. In this study, we propose a novel therapeutic approach based on anti-CXCR4 blocking antibody in combination with NKAE cell therapy to prevent rhabdomyosarcoma tumor implantation and lung metastasis. These results provide the first evidence for the efficacy of this combined immunotherapy for preventing sarcoma disease dissemination.

## Introduction

Sarcomas principally affect children and young adults. Almost half the children with solid tumors, such as rhabdomyosarcoma, osteosarcoma and Ewing sarcoma, have progressive disease despite aggressive treatment. The prognosis is particularly poor for patients with metastatic disease, in at least two thirds of whom the disease progresses (1–3). New therapeutic approaches bypassing the cellular mechanisms of drug resistance are therefore urgently required, particularly for patients with high-risk features.

Chemokines interacting with the chemokine receptors expressed by metastasizing tumor cells play a crucial role in directing the migrating cells to secondary organs. This is well documented for the chemokine CXCL12 and CXC chemokine receptor 4 (CXCR4). Müller et al. discovered a CXCL12–CXCR4 homing axis in cancer metastasis and demonstrated the preferential migration of CXCR4-expressing breast cancer cells toward protein extracts from the lung, which has high levels of CXCL12 expression (4).

CXCL12 binding to CXCR4 stimulates intracellular calcium flux, activates the AKT and ERK signaling pathways, and upregulates the formation of focal adhesions, ultimately increasing migration along gradients of locally expressed and secreted chemokines (5). It also leads to the transcription of genes promoting cell survival and proliferation. CXCR4 is found in various tissues and is predominantly expressed on hematopoietic cells, including B and T cells, monocytes, macrophages, natural killer (NK) and dendritic cells, and on CD34^+^ bone marrow progenitor cells (6). CXCR4 is also weakly expressed on endothelial and epithelial cells, astrocytes and neurons (7, 8).

Khune et al. (Bristol-Myers Squibb) recently developed a fully human monoclonal antibody (mAb), MDX1338/ulocuplumab, specific for human CXCR4 (9). MDX1338 is a high-affinity mAb that blocks CXCL12 binding to CXCR4, thereby inhibiting the cell proliferation and migration promoted by this binding. MDX1338 treatment has been shown to induce apoptosis *in vitro* and anti-tumor activity *in vivo* in hematologic cancers. MDX1338 is an IgG4 isotype mAb. It does not, therefore, mediate antibody-dependent cellular cytotoxicity (ADCC). Its pro-apoptotic effect is associated with the production of reactive oxygen species and does not require caspase activation, as described in a chronic lymphocytic leukemia model (10).

Immune cells are known to recognize and kill tumor cells, suggesting that they could be used in anticancer treatment. NK cells kill cancer stem cells and genotoxically altered cells with a high degree of precision but are tolerant to healthy cells. These properties suggest that they might make good therapeutic effectors for all cancer forms, including metastases (11). Fujisaki et al. (12) and Imai and Iwamoto (13) reported that the K562 leukemia cell line genetically modified to express membrane-bound interleukin (IL)-15 and 4.1-BB ligand (K562-mb15-41BBL) specifically activates human NK cells, driving them into the cell cycle to generate highly cytotoxic NK cells. Furthermore, the K562-mb15-41BBL stimulation method has already been scaled up to large-scale clinical-grade conditions in accordance with good clinical manufacturing practice guidelines, for the production of large numbers of highly cytotoxic NKAE cells, and FDA-approved clinical trials are already underway in patients with hematologic cancers and childhood solid tumors.

Our team has leaded five clinical trials using adoptive NK cell immunotherapy in patients with pediatric refractory solid tumors (NCT01337544), relapsed or refractory acute leukemia/lymphoma (NCT01944982 and NCT02074657), acute myeloid leukemia (NCT02763475) or multiple myeloma (NCT02481934). Our results show it is a promising and feasible therapy (14–16). Based on our experience with NKAE cell therapy, we hypothesized that the anti-metastatic MDX1338 mAb and cytotoxic NK cell therapy would have complementary effects, thereby increasing the efficacy of the therapeutic strategy.

We analyzed CXCR4 expression in tumor samples from pediatric rhabdomyosarcoma patients. We present preclinical data showing the ability of NKAE cell therapy to kill sarcoma cells and of MDX1338 mAb to inhibit the migration and invasion of CXCR4^+^ sarcoma cells *in vitro*. We also used an orthotopic model of alveolar rhabdomyosarcoma to evaluate the complementary antitumoral and antimetastatic effects of combined NKAE + MDX1338 immunotherapy *in vivo*.

## Materials and Methods

### Determination of CXCR4 Expression on Patients' Samples by Immunohistochemistry

Human samples were obtained after informed patient consent in accordance with the Helsinki Declaration. Research was approved by the La Paz Hospital Ethics Committee (PI-2295).

For tumor CXCR4 immunohistochemistry, formalin-fixed, paraffin-embedded tumor slides were deparaffinized, hydrated, stained using anti human CXCR4 mAb (dilution 1:10; clone 44716; R&D systems), visualization system EnVision FLEX+ (mouse, high pH, Agilent) and hematoxylin counterstained. Specificity of the antibody was confirmed by immunostaining of tonsil tissue. The percentage of CXCR4^+^ tumor cells was assigned a score between 0 and 5, and the staining intensity a score between 0 and 3. These 2 values were added. Both cytoplasmic and nuclear staining were assessed and equally weighted to produce the final staining score.

### Cell Culture

143B (CRL-8303), MG-63 (CRL-1427) and A673 (CRL1598) cell lines were from the American Type Culture Collection. RH30 (ACC-489) was from the Leibniz-Institut DSMZ. A4573 and CW9019 were kind gifts from Dr. Javier Alonso (Institute of Health Carlos III) and Dr. Josep Roma (Vall d'Hebron Research Institute), respectively.

Bioluminescent RH30 cells (RH30-GFP-Luc) were generated by infection with a recombinant lentivirus encoding green fluorescent protein (GFP) and firefly luciferase (Luc) under EF1a promoter and with Neomycin marker (Amsbio). Infected cells expressing high GFP levels were isolated by fluorescence activated cell sorting, cloned, expanded, and used for *in vivo* bioluminescence assays. RH30-GFP-Luc cell growth kinetics was similar to parental RH30 cells and they retained surface CXCR4 expression.

Cells were cultured in Dulbecco's modified Eagle's medium (DMEM, Lonza) supplemented with 10% heat-inactivated fetal bovine serum (FBS, Gibco), 2 mM L-glutamine, 50 U/ml penicillin, and 50 μg/ml streptomycin (complete medium).

### NK Cells Activation and Expansion

Peripheral blood mononuclear cells (PBMCs) from healthy donors were isolated by centrifugation over a density gradient (Ficoll-Paque, GE Healthcare) (400 g, 30 min).

The genetically modified K562-mbIL15-41BBL cell line, kindly provided by Professor D. Campana (National University of Singapore) was irradiated with 100 Gy.

NKAE cells were obtained by co-culturing donor's PBMCs with irradiated K562-mb15-41BBL cells in a 1:1.5 ratio plus 100 U/ml of IL-2 (Miltenyi) over 14–21 days in stem cell growth medium (SCGM, Cellgenix) supplemented with 10% human AB serum (Sigma). Fresh medium was added every 2–3 days to a final concentration of 1 × 10^6^ cells/ml. Percentage and phenotype of NK cells (CD3^−^, CD56^+^), T cells (CD3^+^, CD56^−^) and NKT cells (CD3^+^, CD56^+^) was weekly monitored by flow cytometry (Navios, Beckman Coulter) (Supplementary Figure 1). A list of labeled antibodies used in this study is provided in Supplementary Table 1.

### Determination of CXCR4 Expression on Sarcoma Cell Lines by Flow Cytometry and qRT-PCR

For staining, 2 × 10^5^ cells/well were centrifuged in V-bottom 96-well plates and washed with phosphate-buffered saline (PBS, Lonza) containing 0.5% bovine serum albumin (Lonza), 1% FBS and 0.1% sodium azide. Non-specific binding was blocked by pre-incubating the cells with 40 μg/ml rat IgG (Sigma; 100 μl final volume, 20 min, 4°C). Cells were incubated with anti CXCR4-APC mAb (BD Pharmingen; clone 12G5) or isotype matched mAb (30 min, 4°C). Samples were analyzed on a Navios cytometer (Beckman Coulter).

For qRT-PCR, cell culture RNA was obtained using RNeasy Mini Kit (Qiagen) according to the manufacturer's protocol. The relative expression level of mRNA encoding human CXCR4 was determined by quantitative RT-PCR using GUS gene expression as internal control. cDNA was synthesized from 1 μg of total RNA with the Superscript IV First-Strand Synthesis System (Invitrogen). The cDNA was amplified in duplicate with primers for human CXCR4 (Hs00237052_m1, Applied Biosystems) and for human GUS (Fw: 5′-GAAAATATGTGGTTGGAGAGCTCATT−3′, Rv: 5′-CCGAGTGAAGATCCCCTTTTTA−3′; Probe: 5′-[6FAM] CCAGCACTCTCGTCGGTGACTGTTCA[TAMRA]−3′; all from Sigma). Amplification (1 cycle: 50°C for 2 min, 95°C for 10 min; 50 cycles: 95°C for 15 s, 60°C for 1 min) was monitored using the Roche LightCycler 480. Relative expression was analyzed using 2^−ΔCT^ method, where ΔCT = (Ct gene of interest- Ct internal control).

### Cell-Mediated Cytotoxicity Assays

NKAE and RH30-GFP-Luc target cells were co-cultured (4 h) at indicated ratios in DMEM- 10% FBS, then stained with 7-AAD (10 min, 4°C) and analyzed by flow cytometry. Gating on 7-AAD^+^ cells within the GFP^+^ population indicated the proportion of dead target cells. Specific killing was calculated as 100 × [(% dead target cells in sample–% spontaneous dead target cells)/(100–% spontaneous dead target cells)]. Target cells incubated without effector cells were used to assess spontaneous cell death. In Supplementary Figure 2, RH30-GFP-Luc cells were pre-incubated with MDX1338 or isotype IgG4 control mAb (30 min, 100 μg/ml) prior to the assay.

### Cell Migration and Invasion Assays

Cells were incubated in starving buffer (DMEM-1% FBS) for 24 h. Next, 2 × 10^5^ cells were suspended in 80 μl of migration buffer (DMEM-1% human serum albumin, Grifols) and placed in the upper compartment of 96-well transwell plates (8.0 μm pore size, Neuroprobe). The lower compartment was filled with 300 μl of: migration buffer alone (unconditioned medium), migration buffer with 100 ng/ml CXCL12 (R&D systems) or migration buffer with 10% FBS. In invasion assays, the upper compartment of the membrane was coated with 20 μl of 0.2 mg/ml Matrigel (Corning).

After 48 h of incubation to allow cells to migrate/invade through the membrane, the cells remaining on the upper face of the membrane were removed using a cotton wool swab, while the cells on the lower side of the membrane were fixed with methanol and stained with crystal violet. After washing with PBS, the number of migrating/invading cells was determined using a Carl Zeiss Axiovert 200 microscope. Four fields per well were counted at 200x.

For migration/invasion neutralization experiments, 10^5^ RH30 cells were mixed with the indicated final concentration of anti CXCR4 MDX1338 mAb or control IgG4 mAb in a final volume of 90 μl of migration buffer and proceeded as above indicated. In Supplementary Figure 2, 500.000 NKAE cells (effector:target ratio, E:T = 5:1) were also added in the upper well.

### Xenograft Assays

NSG mice (NOD.Cg-Prkdc^scid^ Il2rg^tm1Wjl^/SzJ, Charles River Laboratories) were maintained in the Alberto Sols Biomedical Research Institute animal facility. All procedures were approved by Comunidad de Madrid Animal Protection Area (PROEX 220/16) and CSIC Ethics Committee.

For the *in vivo* metastasis model, 6 weeks of age female mice were inoculated intravenously (i.v.) with 0.5 × 10^6^ RH30-GFP-Luc cells on day 0. Five treatment groups were established (5 mice/group): untreated; isotype control IgG4 mAb; anti CXCR4 MDX1338 mAb; NKAE cells; and MDX1338 + NKAE cells. Antibodies were inoculated intraperitoneally (i.p.), 15 mg/kg, twice a week for 3 weeks. NKAE cells were administered i.v., 5 x 10^6^ cells/mouse, once a week for three weeks.

Luminescent tumors were monitored weekly for 35 days. Briefly, D-luciferin (150 mg/kg; Perkin Elmer) was administered i.p. 10 min before analysis. During imaging, mice were anesthetized with isoflurane in a lightproof chamber using a IVIS-Lumina II (Caliper Lifesciences). Exposure time was 180 s. Living Image v4.5.2 (Perkin Elmer) was used to quantify the data and produce pseudocolor images.

### Histology and Identification of Lung Micrometastases in Mice

Mice dissected organs were fixed overnight in 4% paraformaldehyde (pH 7.4), washed in PBS, paraffin-embedded and cut (3 μm).

The presence of RH30 cells in the lungs was studied by immune staining using anti-human CXCR4 antibody (R&D, clone 44716, dilution 1:50), visualization system EnVision FLEX+ (mouse, high pH; Agilent) and hematoxilin/eosin counterstained.

For *in situ* hybridization, antigen retrieval was first performed with low pH buffer (RiboCC, Roche) and protease III (Roche). Slides were then incubated with the Alu positive control probe II (Ventana, Roche 05272041001). Three stringency washes were performed and slides were incubated with rabbit anti-DNP anti serum. For visualization, OmniRabbit system conjugated with horseradish peroxidase was used (Ventana, Roche). Immunohistochemical reaction was developed using silver as chromogen (Silver kit, Ventana, Roche) and nuclei were counterstained with Carazzi's hematoxylin.

All images were captured on a Carl Zeiss Lab.A1 microscope with an AxioCam ERC5S digital camera (Carl Zeiss).

### qRT-PCR and Quantification of Lung Micrometastases in Mice

One third of mice dissected organs were used for total RNA extraction using RNAeasy Mini kit (Qiagen) according to the manufacturer's protocol. The relative expression levels of mRNA encoding human CXCR4 and human GUS were determined by semi-quantitative RT-PCR with RH30 cells pellet as control of 100% human cells. cDNA synthesis and human CXCR4 and human GUS amplification was performed as above indicated.

### Statistical Analysis

Statistical analyses were performed using GraphPad Prism 7 software. Statistical significance was established at *p* < 0.05 as evaluated by unpaired Student's *t*-test or ANOVA test. Results are shown as mean ± SEM, unless otherwise indicated.

## Results

### CXCR4 Is More Strongly Expressed on Relapsed Samples From Pediatric Rhabdomyosarcoma Patients Than in Diagnostic Samples

**Figure 1:**
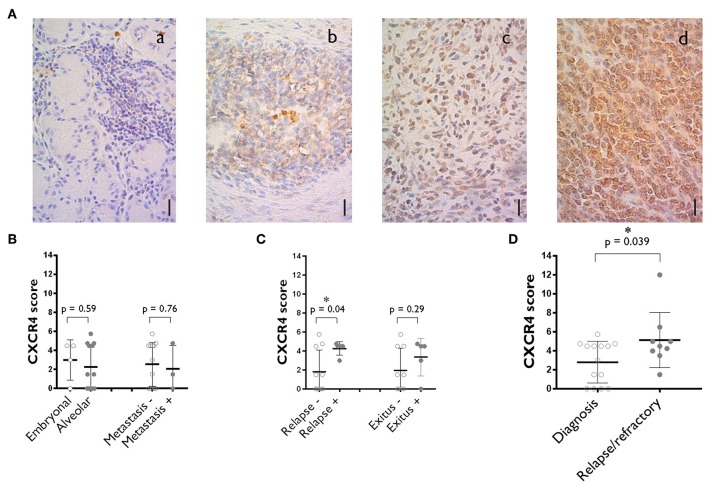
CXCR4 expression on the tumors of rhabdomyosarcoma patients. **(A)** Representative staining corresponding to samples with negative (a), medium (b) and high (c) levels of cytoplasmic CXCR4 expression. A specimen with a high level of nuclear CXCR4 staining is also shown (d). Scale bar = 10 μm. *N* = 26. **(B)** CXCR4 expression score plot for primary tumors at diagnosis where alveolar vs. embryonal rhabdomyosarcomas are compared and where localized vs. already metastasized rhabdomyosarcomas are compared. **(C)** Primary tumors at diagnosis CXCR4 expression vs. outcome (disease relapse or patient's death). **(D)** Comparison of CXCR4 expression between specimens obtained at diagnosis and after chemotherapy (refractory) and during relapse. The values shown are the median ± SD. Unpaired Student's *t*-tests were performed. *p < 0.05.

New treatment options are urgently required for patients whose tumors do not respond to chemotherapy and for relapses after surgery. We investigated the potential role of CXCR4 in chemotherapy resistance and relapse, by performing immunohistochemical analyses to evaluate CXCR4 expression in rhabdomyosarcoma tumor samples. Patients' characteristics are listed in Supplementary Table 2. We analyzed 26 pediatric rhabdomyosarcoma specimens archived in the biobank of La Paz University Hospital from 2010 to 2017. These specimens were taken from patients with a median age of 6.6 years (range: 0.2–14.1) and a median follow-up of 7.9 years (range: 0.6–18.4). Seventeen of the specimens were obtained at diagnosis, five are refractory samples collected after chemotherapy and four specimens corresponded to relapses. As recent studies have reported CXCR4 to be expressed in the nucleus in some cancers (17, 18), we performed both cytoplasmic and nuclear staining, with equal weighting given to the two compartments during the analysis. Cytoplasmic staining for CXCR4 was observed on 68% of the specimens in our series and nuclear staining was observed in 15% (Figure 1A).

For diagnostic samples, no difference in CXCR4 expression was observed between embryonal and alveolar rhabdomyosarcomas or between specimens from localized tumors and those from primary tumors that already had metastasized (Figure 1B). A relationship was observed between CXCR4 score and patient outcome, with higher CXCR4 median score obtained for specimens from patients subsequently suffering from relapses or dying from the disease (Figure 1C).

Moreover, a comparison of CXCR4 expression score between diagnostic (2.8 ± 2.1) and relapsed/refractory (5.1 ± 2.9)specimens revealed a significant trend toward higher levels of CXCR4 expression in relapse samples than in diagnostic samples (Figure 1D).

### The RH30 Alveolar Rhabdomyosarcoma Cell Line Had the Highest Levels of CXCR4 Expression, Concomitant With Highest Migration and Invasion Rates Toward CXCL12

**Figure 2:**
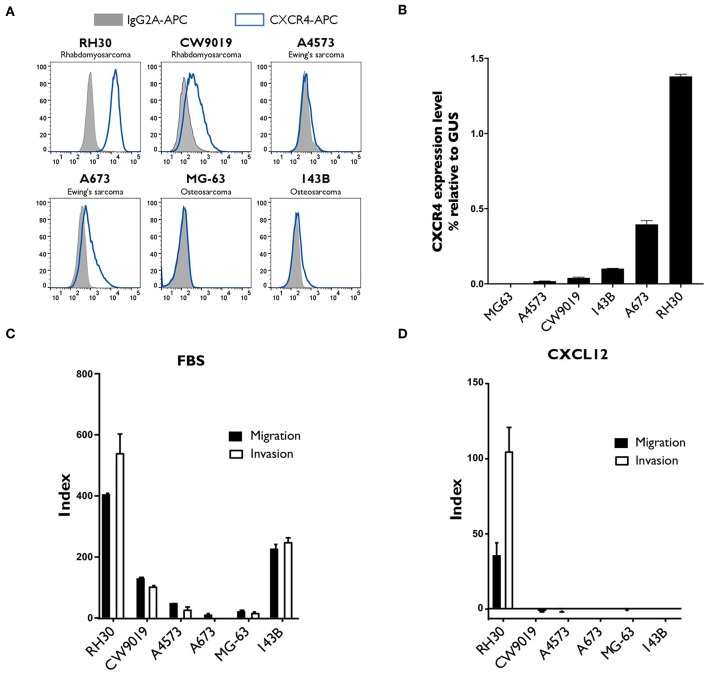
Analysis of CXCR4 expression, and of the migration and invasion capacity of different sarcoma cell lines. **(A,B)** CXCR4 expression was strongest in the RH30 alveolar rhabdomyosarcoma cell line. CXCR4 expression by two rhabdomyosarcoma cell lines (RH30 and CW9019), two Ewing's sarcoma cell lines (A4573 and A673) and two osteosarcoma cell lines (MG-63 and 143B) was analyzed by flow cytometry **(A)** and by RT-qPCR (B). *N* = 3. **(C,D)** RH30 cells migrate and invade along a gradient toward CXCL12 chemokine, CXCR4 specific ligand. Their ability to migrate toward FBS **(C)** or CXCL12 **(D)** was assessed in Transwell assays, with membranes with 8 μm pores. Invasion capacity was measured under the same conditions, with Matrigel-coated Transwell membranes. Each condition was performed in duplicated wells. Representative results of one experiment out of three performed.

We analyzed the surface expression of CXCR4 on confluent cultures of various sarcoma cell lines: RH30, CW9019, A4573, A673, MG-63, and 143B. The proportion of CXCR4^+^ cells was highest in RH30 cell cultures, in which CXCR4 expression was detected on 98.6 ± 0.7% of the cells (Figure 2A). These results were consistent with the analysis of CXCR4 mRNA levels by RT-qPCR analysis. RH30 cultures displayed 1.38 ± 0.01 times higher levels of CXCR4 than of a control gene expression (beta-glucuronidase, GUS) (Figure 2B).

We evaluated the migration and invasion capacity of sarcoma cell lines in Transwell assays, which are considered to provide an *in vitro* indication of metastatic potential *in vivo*. Several sarcoma cell lines migrated toward chemoattractant FBS and displayed invasion capacity (Figure 2C), but only the RH30 cell line migrated and invaded specifically toward the CXCR4 ligand, CXCL12 (Figure 2D). The indices of RH30 migration and invasion toward CXCL12 were 35.2 ± 8.3 and 104.0 ± 16.4, respectively.

### NKAE Cells Were Highly Cytotoxic to RH30 Cells, and MDX1338 Efficiently Reduced RH30 Cell Migration and Invasion Toward CXCL12

**Figure 3:**
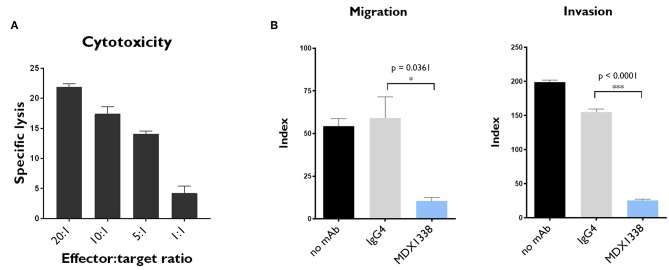
*In vitro* NKAE-mediated cytotoxicity and MDX1338-mediated inhibition of the migration and invasion capacity of rhabdomyosarcoma cells. **(A)** NKAE cells were highly cytotoxic to RH30 cells. Specific lysis was determined at the indicated NKAE:RH30 E:T ratios. **(B)** MDX1338 efficiently decreased the migration and invasion of RH30 cells toward CXCL12. RH30 cell migration was assessed with Transwell plates. Invasion capacity was measured under the same conditions, with Matrigel-coated Transwell membranes. Every condition was performed in duplicates. One representative experiment is shown. *N* = 3. One-way ANOVA test. *p < 0.05, ***p < 0.0001.

NK cells play an important role in the natural control of tumor cells. By co-culturing these cells with the genetically modified cell line K562-mb15-41BBL, large numbers of activated NK cells (NKAE cells) can be obtained from healthy donors. We measured the cytolytic effects of NKAE cells on RH30 sarcoma cells. NKAE cells induced 21.9 ± 0.4% specific lysis of RH30 cells, at an effector:target cell (E:T) ratio of 20:1 (Figure 3A). As expected, MDX1338 had no significant effect on the observed cytotoxicity (Supplementary Figure 2A). MDX1338 is a fully human IgG4 anti-CXCR4 mAb (9, 10), and would not, therefore, be expected to induce ADCC (19).

The effect of MDX1338 on the CXCR4/CXCL12 axis was assessed in RH30 cells, in migration, and invasion assays *in vitro*. MDX1338 significantly decreased the migration of RH30 cells toward CXCL12 and their invasion capacity: 54.4 ± 4.4 vs. 10.5 ± 2.0 for migration and 199.1 ± 2.9 vs. 25.6 ± 1.9 for invasion (Figure 3B). The combination of MDX1338 and NKAE cells totally abolished the migration and invasion capacities of these cells (Supplementary Figure 2B).

### NKAE Cell Treatment Completely Inhibited the Implantation of RH30 Tumor Cells *in vivo*

**Figure 4:**
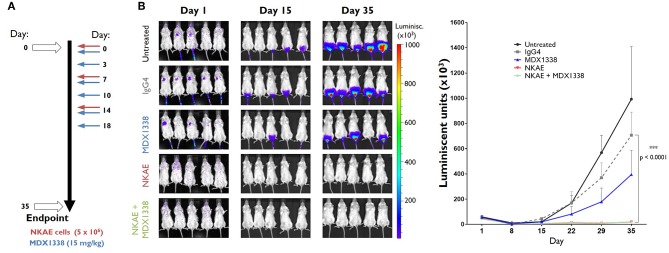
Inhibition of tumor implantation *in vivo* by NKAE cells. **(A)** Administration schedule for the antibody and NKAE cells in NSG mice bearing RH30 GFP^+^ Luc^+^ tumor cells injected intravenously. Five treatment arms were established: untreated; IgG4; MDX1338; NKAE; MDX1338+NKAE (5 mice/group). Two-way ANOVA test. Mice received three doses of NKAE and six doses of mAb. Luminiscent tumors were monitored for 35 days. **(B)** The NKAE treatment completely prevented the peritoneal implantation of RH30 tumors in mice. ***p < 0.0001.

We assessed the antitumor potential of MDX1338 and NKAE cell therapies, by generating an *in vivo* model of metastatic sarcoma, using CXCR4^+^ RH30 cells, which grow as peritoneal xenografts when implanted in immunodeficient NSG mice. Mice were inoculated intravenously with RH30-GFP-Luc cells (day 0). They then received one of the following treatments: MDX1338 mAb, isotype control IgG4 mAb, NKAE cells or combined MDX1338-NKAE cells treatment (Figure 4A). The size of the developing tumors was assessed by measuring the luminescent signal, until day 36, when the mice were killed. NKAE cell therapy alone was sufficient to prevent the intraperitoneal implantation of RH30 tumors entirely (Figure 4B). Mice treated with MDX1338 mAb alone developed intraperitoneal tumors slightly smaller than those of the control mice, whereas a control IgG4 mAb had no significant effect.

### Rhabdomyosarcoma Lung Micrometastases Were Abolished by MDX1338 + NKAE Cell Treatment in an *in vivo* Model

The primary peritoneal RH30 tumors were easy to monitor with the luminescent detection system, but the sensitivity of this approach was too low to determine whether RH30 GFP^+^ Luc^+^ micrometastases were present in distal organs. We therefore used RT-qPCR to assess the presence of human cells by amplifying both human GUS and human CXCR4 cDNAs from the lungs, liver, spleen and bone marrow of the mice. Human gene expression was detected only in the lungs, indicating the presence of RH30 cells in this organ.

**Figure 5:**
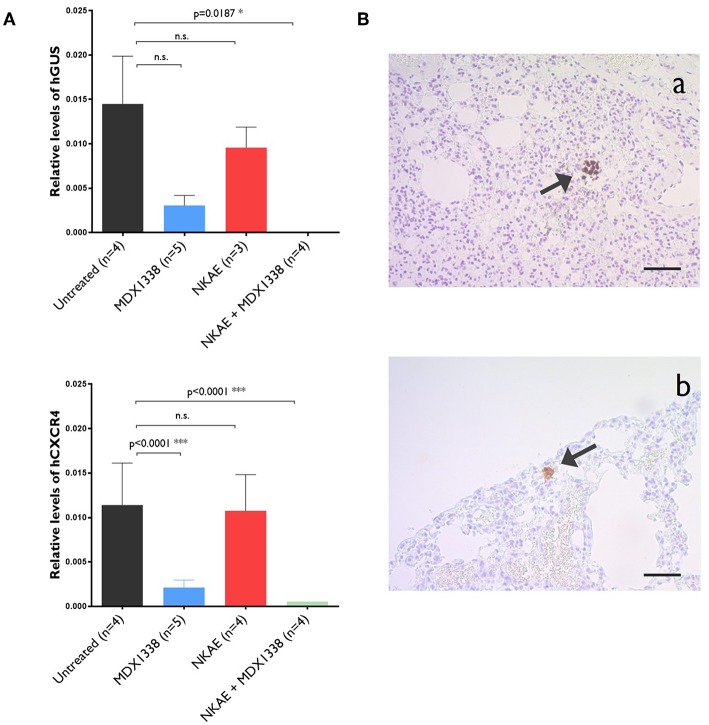
Abolition of the formation of lung micrometastases of rhabdomyosarcoma by MDX1338. **(A)** NKAE-treated mice develop RH30 micrometastases in the lung, but this is entirely prevented by the combination of MDX1338 and NKAE. Micrometastases were detected and quantified by RT-qPCR with a human GUS- or human CXCR4-specific probe. Each sample was analyzed in duplicates, 5 mice per group were analyzed. Two-way ANOVA test. **(B)** The presenceof rhabdomyosarcoma micrometastases was confirmed by histological methods. Lung micrometastases in untreated mice were identified by Alu sequence hybridization (a) and CXCR4-specific mAb staining (b). Scale bar = 20 μm. *p < 0.05, ***p < 0.001.

NKAE cell treatment alone was insufficient to prevent RH30 lung micrometastases. The relative level of expression for human GUS was 14.5 × 10^−3^ ± 5.4 × 10^−3^ in untreated mice and 8.3 × 10^−3^ ± 2.7 × 10^−3^ in NKAE-treated mice. For human CXCR4, relative expression levels were 11.4 × 10^−3^ ± 4.7 × 10^−3^ in untreated mice and 10.7 × 10^−3^ ± 4.1 × 10^−3^ in NKAE-treated mice. MDX1338 treatment alone significantly decreased the formation of micrometastases, but only the combination of MDX1338 and NKAE treatments led to undetectable levels of both human GUS and CXCR4 gene expression in the mouse lungs analyzed (Figure 5A). We have performed a RT-PCR to confirm that the human cells present in the mice lungs were RH30 cells. RH30 cells have *t*_(2, 13)_ chromosomal translocation resulting in PAX3-FOXO1 fusion protein (20). We detected specific chimeric gene transcript PAX3-FOXO1 (21), in untreated, IgG4-, MDX1338- and NKAE-treated mice, whereas we could not detect it in NKAE+MDX1338-treated mice lungs (Supplementary Figure 3).

For further confirmation of the presence of RH30 micrometastases, lung samples were stained with an Alu sequence probe or the anti-human CXCR4 mAb. Human cell aggregates were found in the lungs of untreated mice for both staining procedures (Figure 5B).

## Discussion

Sarcomas, such as Ewing's sarcoma, osteosarcoma, and soft tissue sarcomas like rhabdomyosarcomas, form a group of relatively rare mesenchyme-derived tumors (22). They account for only a small percentage of human cancers overall, but are the second most prevalent solid tumor in children. Despite advances in adjuvant chemotherapy and surgical wide resection, prognosis remains poor, mostly due to the high frequency of lung metastasis, the leading cause of death in these patients.

Chemokines and their interaction with their receptors have been shown to play a fundamental role in the mechanisms of cancer development and metastasis (23). The expression levels of chemokines and their receptors are altered in malignant cells. This has been shown for CXCR4, the chemokine receptor most frequently overexpressed in tumor cells. The interaction of CXCR4 with its ligand, CXCL12, activates a cascade of cellular signaling promoting the survival, proliferation, adhesion, and migration of the CXCR4-expressing tumor cells. This may lead to a relapse of the primary tumor, and it increases the likelihood of distant metastases in organs in which the ligand is secreted, such as the bone marrow and lungs in particular (5, 24).

Aberrant CXCR4 expression has been observed in more than 23 human cancers. The prognostic significance of CXCR4 has been studied in detail in patients with bone and soft tissue sarcomas (25–30). In a recent meta-analysis of 12 studies including a total of 997 sarcoma patients, CXCR4 expression was found to be significantly associated with poor overall survival, higher rates of metastasis and higher tumor stage (18). In our cohort of pediatric rhabdomyosarcoma patients, we found a correlation between higher levels of CXCR4 expression at diagnosis and relapse. Tumor-initiating cells (TICs) are a subpopulation of chemoresistant tumor cells that have been shown to cause tumor recurrence and metastasis. One of the hallmarks of these cells is an upregulation of CXCR4 (24). Consistent with this finding, we observed that the tumors that continued to grow after chemotherapy or that relapsed after surgery and chemotherapy were those with the highest CXCR4 levels (Figure 1C). The elimination of TICs is therefore a priority in the development of new modes of treatments. These cells are the target of our combined anti-CXCR4 mAb and NKAE treatment.

The hijacking of the CXCL12/CXCR4 signaling pathways by tumor cells for the purposes of metastasis and protection from apoptosis rapidly identified the blockade of this axis as a potential treatment target for cancer. A pioneering study by Müller et al. (4) demonstrated the *in vivo* relevance of CXCR4 as a target for cancer therapy, linking the expression of CXCR4 in breast carcinomas with their ability to generate regional lymph node and lung metastases. These data were supported by experiments in which a neutralizing anti-human CXCR4 antibody (murine mAb, clone 44717.111) significantly decreased the frequency of lung, inguinal and axillary lymph node metastases. Similar results were subsequently obtained with another murine anti-human CXCR4 antibody (clone 12G5) (31–33). In a human intratibial osteosarcoma xenograft model, the 12G5 mAb reduced metastatic spread to the lung (34). Interestingly, in a rhabdomyosarcoma xenograft model, a third murine anti CXCR4 mAb (clone CF172) showed some resistance to lymph node metastasis while had little effect on the primary tumor volume (35). Consistent with these previous findings, our results for MDX1338, a fully human mAb blocking the CXCR4/CXCL12 axis, show that this mAb can efficiently decrease the migration and invasion indices of metastatic RH30 rhabdomyosarcoma cells *in vitro*. Similarly, *in vivo*, the combination of MDX1338 and NKAE treatments not only abolished implantation of the RH30 tumor, but also its ability to disseminate, thereby preventing the formation of lung micrometastases.

In physiological non-pathological conditions, CXCL12, and CXCR4 are important for the development of the central nervous system, the heart and the immune system (36, 37). It will therefore be essential to monitor potential adverse effects very carefuly in any therapeutic approach involving a blockade of this axis. Plerixafor, a small-molecule antagonist of CXCR4, is widely used to mobilize hematopoietic stem cells for autologous transplantation in non-Hodgkin's lymphoma and multiple myeloma (38). No serious adverse effects related to CXCL12/CXCR4 blockade have been reported following the administration of plerixafor in patients. MDX1338 has already been tested in phase I and II clinical trials for the treatment of Waldenstrom's macroglobulinemia, leukemia, solid tumors, acute myelogenous leukemia, diffuse large B-cell leukemia, chronic lymphocytic leukemia, follicular lymphoma, and multiple myeloma. Its antitumor effect is mediated by a mobilization of myeloma cells from the bone marrow or by the direct induction of apoptosis, rather than through cellular cytotoxicity, because it is an IgG4 isotype antibody (9, 10, 39). No serious adverse effects due to CXCL12/CXCR4 blockade by MDX1338 have been reported in these trials, either.

In this study, we propose a novel immunotherapy for the treatment of sarcomas. This treatment combines the ability of NK cells to eliminate tumor cells without prior sensitization with anti-CXCR4 treatment to decrease the incidence of metastasis. We previously showed that osteosarcoma cells are susceptible to NKAE lysis both *in vivo* and *in vitro*, and that this cytolytic activity is dependent on interactions between the NKG2D receptor and its ligands (40). The NKAE cells targeted sarcoma cells, including the TICs compartment. We therefore decided to test the potentially additive effects of MDX1338 and NKAE cell therapy for the treatment of rhabdomyosarcoma. We show here that a combination of NKAE cells and MDX1338 efficiently inhibits rhabdomyosarcoma cell invasion, tumor implantation and metastasis.

As mentioned, NK constitutively express CXCR4 and so do NKAE cells (41). The effect of anti CXCR4 on NKAE should, therefore, be taken into account in the proposed combined therapy. In mice, the administration of the CXCR4 antagonist, AMD-3100/plerixafor to C57BL/6 mice induced strong reduction of NK cells in the bone marrow and increased their number in blood and spleen (42). These results are transposable to our model, since the CXCR4 and CXCL12 genes are remarkably conserved across diverse species. The human and murine CXCL12 differs by one amino acid and is cross reactive (43). Similarly, in a rhesus macaque model, AMD-3100 treatment led to mobilization of NK, together with B and T cell subpopulations into the peripheral blood (44). In our model, we hypothesize that MDX1338 blocks not only metastatic CXCR4^+^ sarcoma cells migration to the lungs, but also blocks therapeutic NKAE cells homing to the bone marrow and favors their permanence in the blood stream, where they are more likely to find and kill metastatic neoplastic cells.

Clinical trials of the adoptive transfer of NK cells have been published. These trials made use of resting NK cells (45, 46) or NK cells cultured with IL-2 (47–49). The use of activated and expanded NK cells cocultured with human-derived antigen-presenting cells, as proposed here, is an emerging alternative (14, 15, 50, 51). Our and others' results indicate that both autologous and haploidentical NKAE cell therapies are safe, with no serious adverse effects, and feasible for use in the treatment of various types of cancer.

The results presented here demonstrate that combined treatment with the anti-CXCR4 mAb MDX1338 and NKAE cell therapy prevents rhabdomyosarcoma cells from migrating, invading, tumor implantation and the formation of lung metastases. They support the establishment of a clinical trial to evaluate this pioneering combined treatment scheme. Previous studies have indicated that both these treatments are well tolerated, at least when used separately, so the translation of this approach into clinical practice should be straightforward. This is an important point given the urgent need for new strategies to improve treatment efficacy in sarcoma patients with metastatic disease.

## Data Availability

All datasets generated for this study are included in the manuscript and/or the Supplementary Files.

## Ethics Statement

Human samples were obtained after informed patient consent in accordance with the Helsinki Declaration. Research was approved by the La Paz Hospital Ethics Committee (PI-2295). All procedures were approved by Comunidad de Madrid Animal Protection Area (PROEX 220/16) and CSIC Ethics Committee.

## Author Contributions

MV and AP-M: conception, design, and writing, review, and/or revision of the manuscript. MV, PG-N, LF, AE, JP-K, and MM: development of methodology. MV, DB, PG-N, AB, JV, CM-D, MA-Á: acquisition of data (provided animals, acquired and managed patients, provided facilities, etc.). MV, DB, PG-N, AB, RP, MM, JP-K, and AP-M: analysis and interpretation of data (e.g., statistical analysis, biostatistics, computational analysis). MV, PG-N, AB, and CM-D: administrative, technical or material support (i.e., reporting or organizing data, constructing databases). AP-M: study supervision.

### Conflict of Interest Statement

The authors declare that the research was conducted in the absence of any commercial or financial relationships that could be construed as a potential conflict of interest.
