# Supplementary material for: Anti-CXCR4 Antibody Combined With Activated and Expanded Natural Killer Cells for Sarcoma Immunotherapy
Source: Front Immunol. 2019 Aug 2;10:1814. doi: 10.3389/fimmu.2019.01814 (PMC6688426; doi:10.3389/fimmu.2019.01814)
Supplement: Supplementary file 1 [file Data_Sheet_1.docx]

Supplementary Material

# Supplementary Figures and Tables

## Supplementary Figures


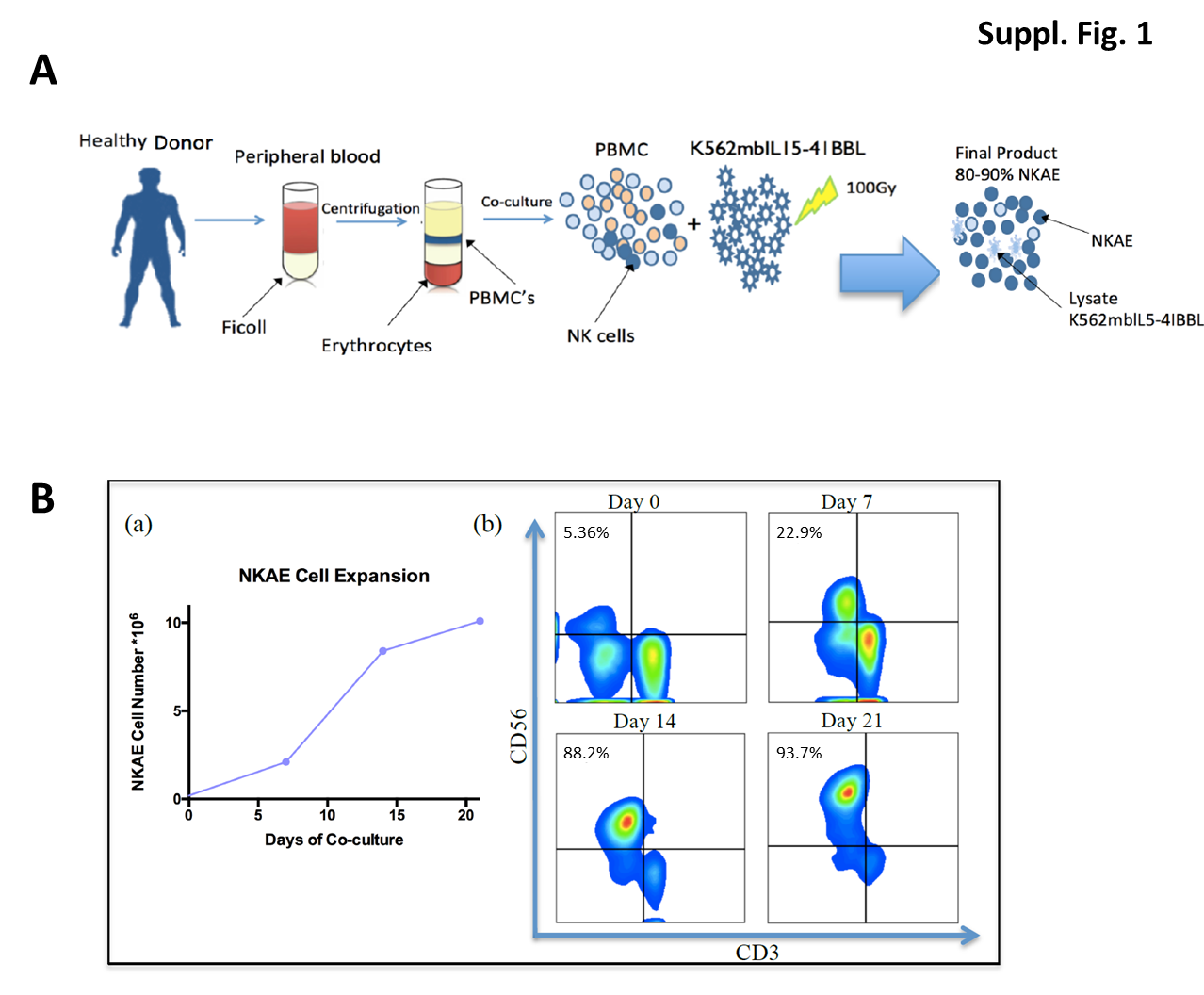


**Supplementary Figure 1. Activation and expansion of NK cells**

**A.** NK cells obtained from the peripheral blood of healthy volunteers are co-cultured for two to three weeks with irradiated K562-mb15-41BBL cells, to obtain large numbers of NKAE cells. **B.** Exponential growth in the number of NK cells (a) and the purity of the preparation (b) over time. The coculture contained 85-90% NKAE cells by day 14.


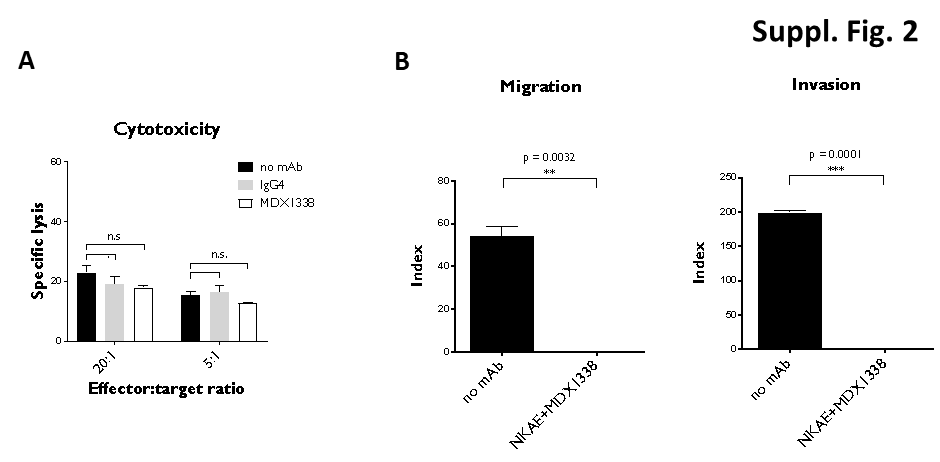


**Supplementary Figure 2. Rhabdomyosarcoma cells cytotoxicity and inhibition of migration and invasion capacity by a combination of NKAE cells and MDX1338 *in vitro***

**A.** MDX1338 did not induce antibody-dependent cellular cytotoxicity in NKAE cells, which retained their capacity to lyse RH30 cells. Specific lysis in the absence of antibody, with IgG4 control or anti-CXCR4 MDX1338 mAb (100 µg/ml) was assessed at the indicated RH30:NKAE E:T ratios. B. The combination of MDX1338 and NKAE cells completely abolished the migration and invasion of RH30 cells towards CXCL12. We assessed the ability of RH30 cells to migrate along a gradient towards human recombinant CXCL12 on Transwell plates. We used an antibody concentration of 100 µg/ml and the RH30:NKAE E:T ratio was 5:1. Invasion capacity was measured in the same conditions, with Matrigel-coated Transwell membranes.

**
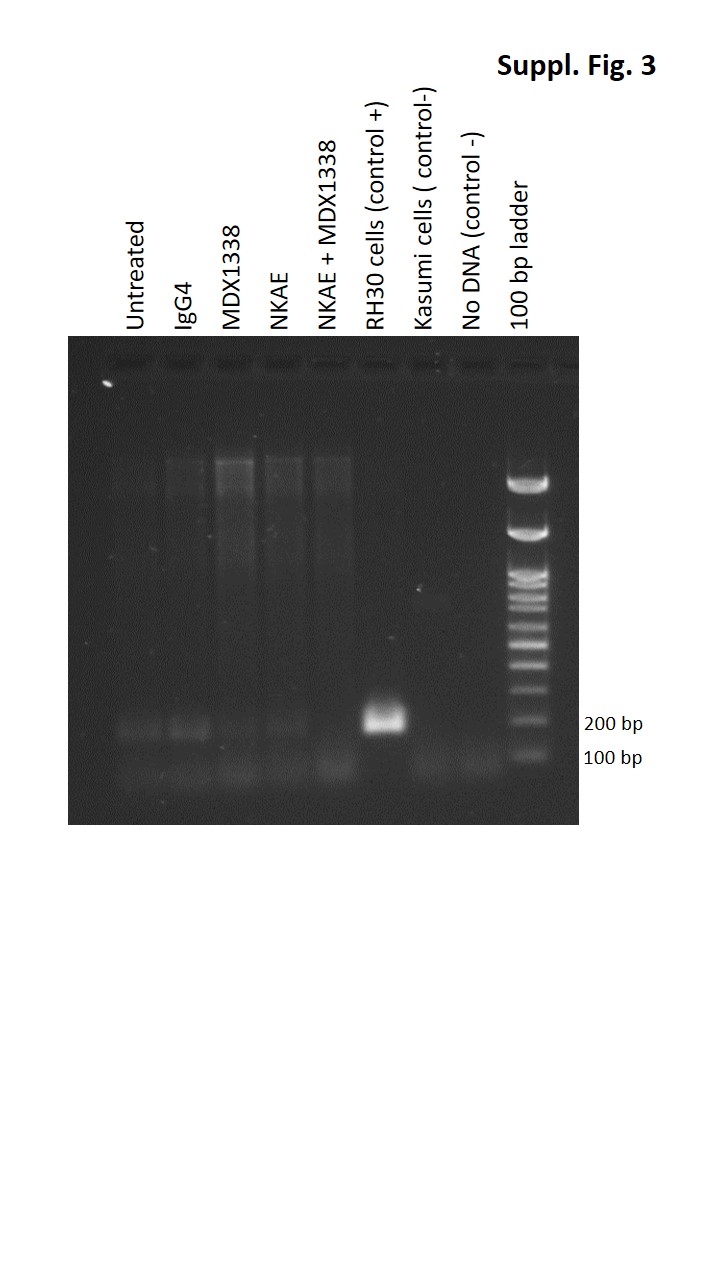
**

**Supplementary Figure 3. RT-PCR to detect specific chimeric gene transcript PAX3-FOXO1, present in RH30 cells and absent in NKAE cells in cDNA from one mice of each treatment group.**

Primers: PAX3/7-a 5’-CAGACAGCAGCTCTGCCTAC-3’; FOXI1 5’-ATGAACTTGCTGTGTA GGGACAG-3’. Programmable temperature cycling was performed using the following profiles: 95°C for 5 minutes, followed by 40 cycles of 94°C for 1 minute, 62°C for 1 minute, and 72°C for 2 minutes. Expected amplicon size in fusion transcript positive samples: 171 bp.

## Supplementary Tables

**Supplementary Table 1.** List of labeled antibodies used in this study.

| **Specificity** | **Clone** | **Isotype** | **Fluorochrome** | **Manufacturer** |
| --- | --- | --- | --- | --- |
| CD3 | UCHT1 | mIgG1 | PE/Cy7 | Biolegend |
| CD45 | J33 | mIgG1 | FITC | Beckman Coulter |
| CD56 | B159 | mIgG1 | APC | BD Bioscience |
| CXCR4 | 12G5 | mIgG2a | APC | BD Biosciences |
| Isotype control | 20102 | mIgG2a | APC | R&D |

**Supplementary Table 2.** Pediatric rhabdomyosarcoma patients’ characteristics and CXCR4 staining score of their samples.

| **Characteristics at diagnosis** | | | | | **Treatment** | | | | **Outcome** | | **CXCR4 expression** | |
| --- | --- | --- | --- | --- | --- | --- | --- | --- | --- | --- | --- | --- |
| **Patient** | **Sex** | **Age at diagnosis (years)** | **Tumor type** | **Metastasis at diagnosis** | **Protocol** | **Treatment group (risk)** | **Response to chemotherapy** | **Post chemotherapy treatment** | **Relapse** | **Exitus** | **Sample type** | **CXCR4 score** |
| **#1** | M | 5.4 | Embryonal | No | SIOP 95 | A | PR | CH+RT | Yes | Yes | D | 4.5 |
| **#2** | M | 11.1 | Alveolar | No | SIOP 95 | B | PR | CH+RT | Yes | Yes | D | 4.5 |
| **#3** | F | 9.9 | Alveolar | NA | NA | NA | NA | NA | NA | NA | D | 0 |
| **#4** | M | 8.5 | Embryonal | Yes | SIOP 95 | A | PR | CH+RT | Yes | Yes | D | 4.75 |
| **#5** | M | 4.5 | Embryonal | No | SIOP 95 | A | PR | CH+RT | No | No | D | 4.75 |
| **#6** | F | 2.4 | Embryonal | No | EpSSG RMS2005 | C (standard) | PR | CH | No | No | D | 5.75 |
| **#7** | F | 7.6 | Alveolar | No | EpSSG RMS2005 | G (high) | PR | CH+RT | Yes | Yes | D | 3 |
| **#8** | M | NA | NA | NA | EpSSG RMS2005 | D (standard) | SD | CH | NA | NA | D | 0 |
| **#9** | F | 6.6 | Undifferentiated | Yes | EpSSG RMS2005 | H (very high) | CR | CH | No | No | D | 0 |
| **#10** | F | 7.1 | Embryonal | No | EpSSG RMS2005 | F (high) | PR | CH+RT | No | No | D | 0 |
|  |  |  |  |  |  |  |  |  |  |  | Post CH | 5 |
| **#11** | F | 6.7 | Embryonal | No | EpSSG RMS2005 | E (high) | PR | CH+RT | No | No | D | 4.5 |
|  |  |  |  |  |  |  |  |  |  |  | Post CH | 4 |
| **#12** | M | 7.5 | Alveolar | Yes | EpSSG RMS2005 | H (very high) | PR | CH+RT | No | No | D | 1.5 |
| **#13** | F | 2.5 | Embryonal | No | EpSSG RMS2005 | D (standard) | PR | CH+RT | No | No | D | 1.5 |
| **#14** | F | 3.9 | Embryonal | No | EpSSG RMS2005 | B (standard) | CR | CH | No | No | D | 0 |
| **#15** | M | 13.0 | Embryonal | No | EpSSG RMS2005 | C (standard) | PR | CH+RT | Yes | No | D | 4.5 |
|  |  |  |  |  |  |  |  |  |  |  | R | 4.5 |
| **#16** | M | 2.0 | Embryonal | No | EpSSG RMS2005 | D (standard) | PR | CH+RT | No | Yes | D | 0 |
| **#17** | F | 1.7 | Embryonal | No | EpSSG RMS2005 | G (high) | PR | CH+RT | No | No | D | 0 |
|  |  |  |  |  | EpSSG RMS2005 |  |  |  |  |  | Post CH | 5 |
| **#18** | M | 3.8 | Embryonal | No | SIOP 95 | A | PR | CH+RT | Yes | Yes | R | 6.5 |
| **#19** | M | 12.9 | Alveolar | No | EpSSG RMS2005 | G (high) | CR | CH+RT | Yes | Yes | R | 12 |
| **#20** | M | 0.2 | Embryonal | No | EpSSG RMS2005 | F (high) | PR | CH+RT | Yes | Yes | R | 1.5 |
| **#21** | F | 4.4 | Embryonal | No | EpSSG RMS2005 | A (low) | PR | CH | No | No | Post CH | 3.5 |
| **#22** | M | 10.6 | Embryonal | No | EpSSG RMS2005 | NA (high) | NA | CH+RT | No | No | Post CH | 4.25 |

**Table abbreviations:** M: Masculine; F: Feminine; SIOP 95: International Society of Paediatric Oncology 95; EpSSG RMS2005: The European paediatric Soft tissue sarcoma Study Group (EpSSG) Rhabdomyosarcoma (RMS) 2005; PR: Partial Response; SD: Stable Disease; CR: Complete Response; CH: Chemotherapy; RT: Radiotherapy; D: Diagnosis; R: Relapse; NA: Not Available.
